# Supplementary material for: Nutritional Calcium Supply Dependent Calcium Balance, Bone Calcification and Calcium Isotope Ratios in Rats
Source: Int J Mol Sci. 2022 Jul 14;23(14):7796. doi: 10.3390/ijms23147796 (PMC9322359; doi:10.3390/ijms23147796)
Supplement: Supplementary file 1 [file ijms-23-07796-s001.zip › ijms-1765624-supplementary.pdf]

Supplements

Supplemental Tables

Table S1. Serum Ca, phosphate, creatinine and alkaline phosphatase were measured weekly in group 1 (n=2-5), group 2 (n=1-5) and group 3 (n=2-4 animals per measurement). Serum Ca, phosphate creatinine, and alkaline phosphatase were in the normal range for rats throughout [49].

	**Baseline** (Group 1)	**Standard-Ca-diet** (Group 2)	**Low-Ca-diet** (Group 3)
Calcium (mmol/l)	2.84 ± 0.03	2.78 ± 0.12	2.70 ± 0.10
Phosphate (mmol/l)	3.9 ± 0.4	2.69 ± 0.6	2.9 ± 0.3
Creatinine (mg/dl)	0.42 ± 0.10	0.27 ± 0.10	0.24 ± 0.06
Alkaline phosphatase (U/l)	472 ± 57	397 ± 150	400 ± 102

Table S2. Tibia μCT studies were performed after 2 weeks of standard-Ca-diet (0.5% Ca; baseline group 1), after a total of six weeks of standard-Ca-diet (group 2) and in rats after two weeks of standard-Ca-diet followed by 4 weeks of a low-Ca-diet (0.25%; group 3).

Right tibia		**Baseline** Week 2 (Group 1; n=10)	**Standard-Ca-diet** Week 6 (Group 2; n=19)	**Low-Ca-diet** Week 6 (Group 3; n=20)	p-value[a]
Volume (mm^3)	Total bone	301 ± 20	406 ± 22	373 ± 24	.0002
Volume (mm^3)	Δ tibia volume[b]		105 ± 22	72.0 ± 24	.0002
Volume (mm^3)	Cortical bone	201 ± 12	305 ± 14	275 ± 15	<.0001
Volume (mm^3)	Cancellous bone	100 ± 15	101 ± 9.6	98 ± 11	.41
Volume (mm^3)	Diaphysis[c]	12 ± 0.4	17.9 ± 1.3	16.9 ± 0.7	.004
Volume (mm^3)	Metaphysis[c]	44 ± 4.2	30.4 ± 2.4	25.5 ± 3.4	<.0001
Volume (mm^3)	Epiphysis[c]	7.6 ± 1.6	14.1 ± 2.8	13.1 ± 1.8	.18
Bone mineral density (HU)	Total bone HU	3791 ± 108	4043 ± 64	3992 ± 73	.03
Bone mineral density (HU)	Δ HU[b]		252 ± 64	201 ± 73	.03
Bone mineral density (HU)	Cortical bone HU	2819 ± 108	3086 ± 64	3041 ± 71	.05
Bone mineral density (HU)	Cancellous bone HU	972 ± 7.0	957 ± 5.5	951 ± 5.1	.002
Bone mineral density (HU)	Diaphysis HU[c]	3655 ± 162	3667 ± 122	3581 ± 101	.02
Bone mineral density (HU)	Metaphysis HU	2586 ± 53	2945 ± 52	2875 ± 67	.001
Bone mineral density (HU)	Epiphysis HU[c]	2714 ± 59	2885 ± 64	2793 ± 50	<.0001
Δ Hounsfield Units × tibia volume (HU × mm^3)			101341 ± 21862	74059 ± 24955	.001

a = G2 vs. G3, t-test; b = in the period of week 3-6; c = cortical + cancellous bone

Table S3. In group 1, six additional female rats were compared to the 12 male animals. All animals received 0.5% Ca diet for 2 weeks. Dietary food and thus Ca intake were reduced in female rats entailing an also reduced net body Ca uptake. None of the Ca isotope ratios in the female rats differed significantly from the male animals of group 1.

			Week 1/2		p-value[a]
			female	male	
Ca balance	Food intake (g/day)		15.4 ± 0.4	22.1 ± 1.0	.001
	Dietary Ca intake (mg/day)		76.8 ± 1.8	111 ± 5.0	.001
	Fluid intake (ml/day)		22.9 ± 0.8	32.2 ± 1.2	<.0001
	Fluid Ca intake (mg/day)		2.5 ± 0.1	3.5 ± 0.1	<.0001
	Feces (g/day)		1.1 ± 1.1	2.8 ± 0.7	.001
	Fecal Ca loss (mg/day)		12.4 ± 6.7	12.9 ± 6.4	.25
	Intestinal Ca absorption (%)		90.5 ± 3.9	88.6 ± 5.6	.65
	Intestinal Ca absorption (mg/day)		71.5 ± 3.1	100.9 ± 6.4	.002
	Urine excretion (ml/day)		8.1 ± 4.0	8.5 ± 3.2	.83
	Urine Ca excretion (mg/day)		1.0 ± 0.7	0.7 ± 0.4	.74
	Net body Ca uptake (mg / day)		70.6 ± 2.7	100.4 ± 6.6	.002
μCT	Volume (mm^3)	Tibia	249 ± 13	301 ± 20	<.0001
		Cortical bone	177 ± 8	201 ± 12	<.0001
		Cancellous bone	71 ± 6	100 ± 15	<.0001
	Bone density (HU)	Tibia	3977 ± 21	3791 ± 108	.001
		Cortical bone	3004 ± 21	2819 ± 108	.001
		Cancellous bone	973 ± 2	972 ± 7	.68
$\delta^{44/42}$Ca (‰)	Bone	Femur	0.11 ± 0.04	0.15 ± 0.05	.34
		Lower ribs	0.14 ± 0.10	0.13 ± 0.05	.86
		Tibia: diaphysis	0.14 ± 0.05	0.18 ± 0.06	.14
		Tibia: metaphysis	0.28 ± 0.05	0.27 ± 0.07	.94
		Tibia: epiphysis	0.20 ± 0.06	0.26 ± 0.04	.11

		Week 1		p-value[a]	Week 2		p-value[a]
		female	male		female	male	
$\delta^{44/42}$Ca (‰)	Serum	0.47 ± 0.07	0.52 ± 0.03	.06	0.56 ± 0.03	0.58 ± 0.05	.54
	Feces	0.78 ± 0.06	0.88 ± 0.17	.39	0.95 ± 0.11	1.04 ± 0.12	.38
	Urine	1.19 ± 0.51	1.16 ± 0.54	.92	1.58 ± 0.21	1.54 ± 0.28	.84
Serum Ca (mmol/l)		3.06 ± 2.36	2.86 ± 0.73	.83	2.67 ± 0.27	2.82 ± 0.36	.87

a = t-test

Supplemental Figures

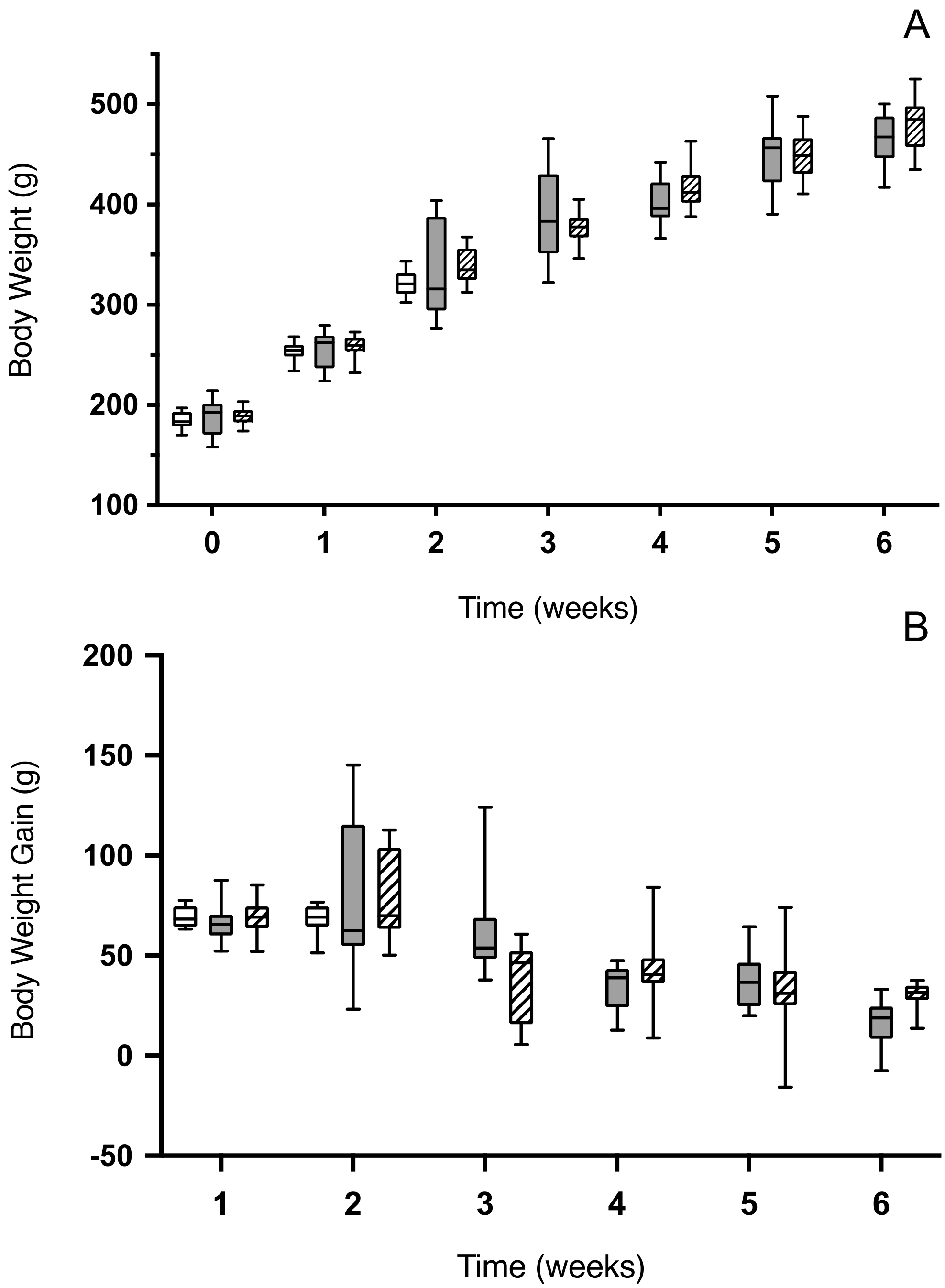

Figure S1. Body weight and body weight gain Body weight (A) and weekly body weight gain (B) was similar in animals of group 1 (empty boxes), group 2 on standard-Ca-diet (gray boxes) and group 3 on low-Ca-diet (hatched boxes) throughout the study.

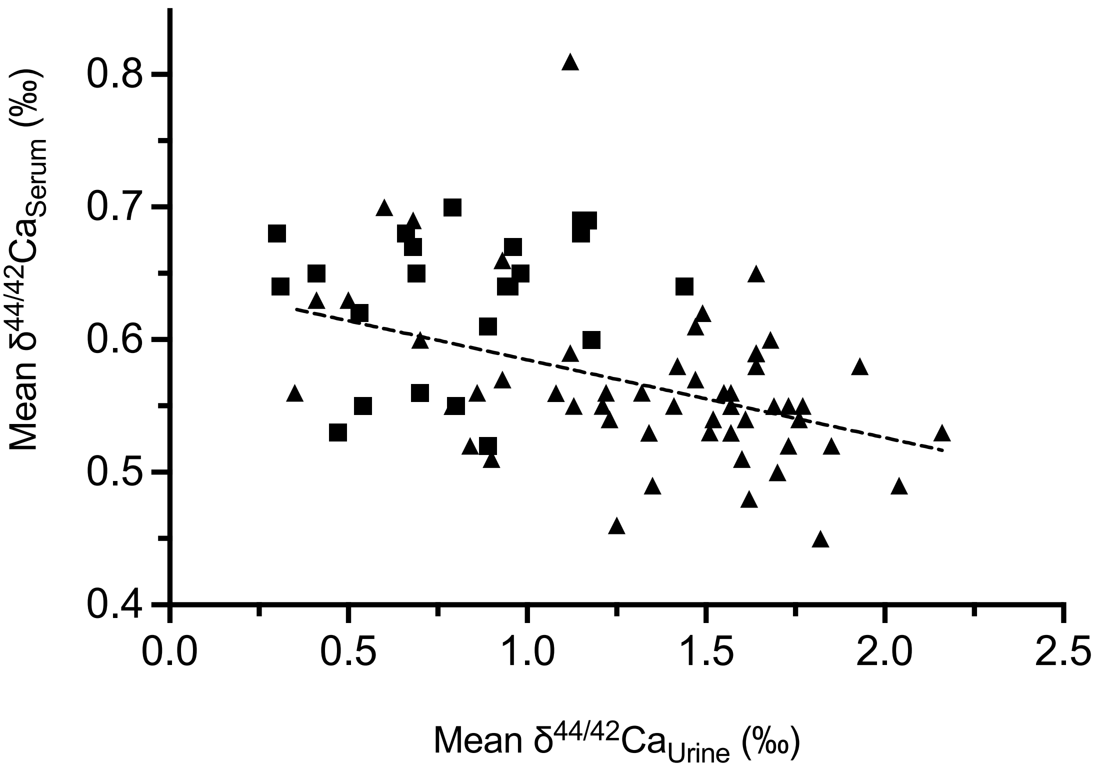

Figure S2. Mean $\delta^{44/42}Ca_{Urine}$ and $\delta^{44/42}Ca_{Serum}$ in rats on low and standard-Ca-diet Two weeks means of $\delta^{44/42}Ca_{Urine}$ in rats on standard-diet (triangles) and low-Ca-diet (squares) are shown relative to the respective mean $\delta^{44/42}Ca_{Serum}$. In animals on standard-Ca-diet $\delta^{44/42}Ca_{Urine}$ inversely correlated with $\delta^{44/42}Ca_{Serum}$ (r=-0.41, p=0.003; n=53) but not in animals on low-Ca-diet (r=0.18, p=0.39; n=23).
